# Glial plasticity at nervous system transition zones

**DOI:** 10.1242/bio.060037

**Published:** 2023-10-03

**Authors:** Laura Fontenas

**Affiliations:** Department of Biological Sciences, Florida Atlantic University, Jupiter, FL 33458, USA

**Keywords:** Glia, Migration, Motor exit point, Dorsal root entry zone, Nervous system, Transition zone

## Abstract

The central and peripheral nervous systems (CNS and PNS, respectively) are two separate yet connected domains characterized by molecularly distinct cellular components that communicate via specialized structures called transition zones to allow information to travel from the CNS to the periphery, and vice versa. Until recently, nervous system transition zones were thought to be selectively permeable only to axons, and the establishment of the territories occupied by glial cells at these complex regions remained poorly described and not well understood. Recent work now demonstrates that transition zones are occupied by dynamic glial cells and are precisely regulated over the course of nervous system development. This review highlights recent work on glial cell migration in and out of the spinal cord, at motor exit point (MEP) and dorsal root entry zone (DREZ) transition zones, in the physiological and diseased nervous systems. These cells include myelinating glia (oligodendrocyte lineage cells, Schwann cells and motor exit point glia), exit glia, perineurial cells that form the perineurium along spinal nerves, as well as professional and non-professional phagocytes (microglia and neural crest cells).

## Teaser

**Summary:** Until recently, the nervous system boundaries were thought to be permeable only to axons. This review highlights recent work that illustrates glial cell transgression of nervous system transition zones.

## Introduction

Although commonly thought of as two separate domains known as the central nervous system (CNS) and peripheral nervous system (PNS), the vertebrate nervous system functions as one unit.

The CNS is composed of the brain and spinal cord, while the PNS includes ganglia and nerves that connect the CNS to various parts of the body. Bi-directional communication between the CNS and the PNS is essential and is possible at transition zones, specialized regions punching the cover of the spinal cord at regular intervals, where central and peripheral neural tissue intimately meet. Spinal cord transition zones are comprised of motor exit points (MEP) ventrally, and dorsal root entry zones (DREZ), dorsally. These regions are traversed by bundles of nerve fibers as well as central and peripheral non-neuronal components of the nervous system. Motor neurons, located in the ventral spinal cord, project their axons through MEP transition zones in order to innervate their target muscles in the periphery. Conversely, sensory axons that relay information such as touch and pain from the periphery toward the brain, enter the spinal cord at DREZ.

Glial cells, or glia, are an essential non-neuronal constituent of the nervous system. Both in the CNS and in the PNS, glial cells provide neurons and axons with metabolic and trophic support, enable saltatory conduction by producing myelin, but they also shape, remodel, and synchronize neural circuits. Glial cells were originally described as residents of either the CNS or the PNS, that are not capable of freely crossing the CNS/PNS boundaries. The CNS and the PNS are two separate yet connected domains in which distinct cell types often carry out similar biological functions. For example, oligodendrocytes are glial cells restricted to the CNS and Schwann cells are glial cells limited to the PNS, and both glial subtypes function to myelinate axons (Bergles and Richardson, 2015; Salzer, 2015).

During embryonic development, neural crest cells give rise to many structures of the PNS including peripheral glial cells, while the neural tube contributes to structures that build the CNS. There is growing evidence indicating interactions between glial cells residing in distinct domains of the nervous system and demonstrating their transgression of nervous system boundaries in physiological and pathological conditions (Sharma et al., 1995; Vermeren et al., 2003; Kucenas et al., 2008, 2009; Coulpier et al., 2010; Smith et al., 2014, 2016; Fontenas and Kucenas, 2017, 2021; Fontenas et al., 2019; Green et al., 2019). How certain cells freely cross the nervous system boundaries, while some others are strictly restricted to one compartment is a fascinating question that modern techniques are just starting to answer. Understanding how glial cells shape and transgress the nervous system boundaries is essential for promoting neural repair and regeneration as well as developing targeted therapies for various neurological disorders.

## Part I: pioneering studies that shaped the nervous system transition zones

Nervous system transition zones have historically been explored in a wide array of species that include the lamprey, rat, cat, grass hopper, human, and were originally described by using methods such as autoradiography, immunohistochemistry and electron microscopy (Fraher, 1978, 1992; Bastiani and Goodman, 1986; Doucette, 1991; Fraher and Cheong, 1995; O'Brien et al., 2001). These studies revealed that in vertebrates, the structure that delineates the spinal cord is a barrier composed of specialized glial cells, known as astrocytes, creating a glial limiting membrane or glia limitans. This astrocytic barrier has been thought to prevent the mixing of myelinating glial cells that reside in the CNS and PNS during development (Fraher, 1992).

The glia limitans was comprehensively described using *in vitro* preparations of dissected tissue and allowed for a better understanding of CNS/PNS cellular segregation. *In vitro* models of astrocyte/Schwann cell interactions have revealed that astrocytes exclude Schwann cells from the CNS by limiting their migration via N-cadherin-mediated adhesion and that Schwann cells are expelled when transplanted into the glia limitans (Baron-Van Evercooren et al., 1992; Wilby et al., 1999). These glia–glia interaction studies also described irregular interdigitations of Schwann cells and astrocytes at the DREZ and illustrated the non-overlapping territories occupied by astrocytes and peripheral glia (Fraher, 1999).

The origin of peripheral myelinating glia has been subject to controversy for decades, and was even referred to as “a problem” by the embryologist Hörstadius in the mid-twentieth century (Hörstadius, 1950). While it had been accepted that Schwann cells derived from the neural crest, Weston described in the early 1960s the dual origin of “Schwann sheath cells” along spinal motor nerves. He indeed described Schwann sheath cells of neural tube origin associated with ventral nerve fibers in chick embryos lacking neural crest or neural crest derivatives (Weston, 1963). In the same article, Weston reviewed compelling evidence indicating a neural crest origin for Schwann sheath cells and equally convincing evidence suggesting that certain sheath cells found in the periphery came from the neural tube. His findings from crestless neural tube grafts supported the idea that some sheath cells came from the neural tube (Weston, 1963)*.* After a long hiatus, the neural tube origin of ventral root sheath cells was confirmed in the late 1980s. Using quail–chick grafting experiments, Lunn et al. described that following the graft of a crest-ablated quail neural tube in place of a host chick neural tube, a population of quail cells was found at motor exit point transition zones, tightly associated with ventral root axons (Lunn et al., 1987). By corroborating a neural tube origin for a population of ventral root sheath cells, these observations resolved a prolonged dispute and established a new avenue of research focused on understanding glial cell migration across the nervous system boundaries.

## Part II: the emergence of modern techniques and *in vivo* imaging has contributed to a better understanding of nervous system transition zones

Interactions between the CNS and the PNS play an important role in shaping the developing nervous system and remain important in the pathophysiological adult nervous system. Recent studies have shed light on cellular and molecular mechanisms underlying glial cell migration and plasticity at nervous system transition zones.

### The dorsal root entry zone

#### Non-myelinating OLCs adopt sensory oligodendrocyte identity by contacting peripheral sensory axons

**Fig. 1.**
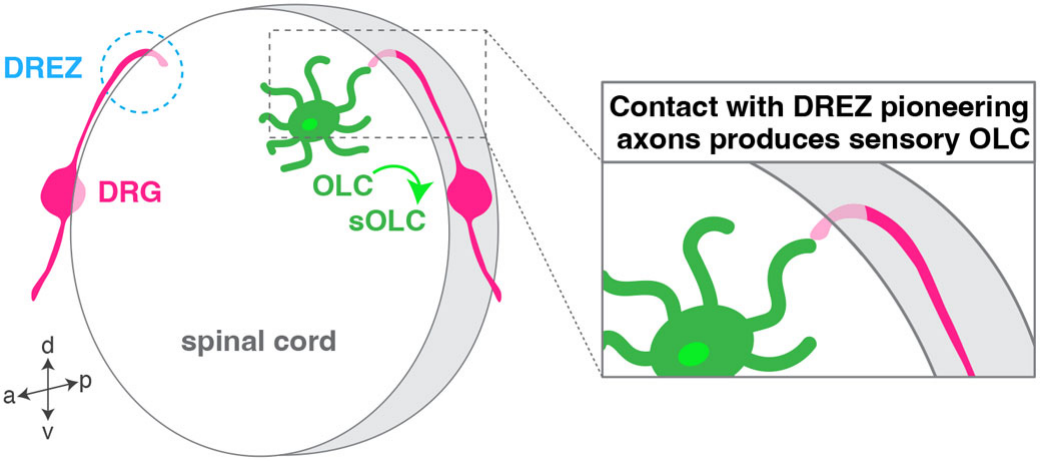
**A subset of oligodendrocyte lineage cells interacts with the developing dorsal root entry zone.** Oligodendrocyte lineage cells (OLC, green) contact pioneering sensory axons (magenta) as they enter the spinal cord at the dorsal root entry zone (DREZ). Upon contact, these OLCs adopt a sensory oligodendrocyte (sOLC) identity.

While *in vitro* and electron microscopy analyses of DREZ cellular components taught us that CNS and PNS tissues do not mix, live imaging now indicates cellular interactions across the nervous system boundaries. By using zebrafish as a model system, Green et al. demonstrated that peripheral components of the DREZ are important to generate diversity within oligodendrocyte lineage cells (OLC) in the spinal cord through contact-mediated communication (Green et al., 2022). In this study, Green et al. showed that pioneering sensory axons growing from the dorsal root ganglia (DRG) interact with CNS-located OLCs as they enter the spinal cord at the DREZ, around 52 h post fertilization, which subsequently generates molecularly distinct sensory OLCs (Fig. 1). Altering DREZ formation by pharmacologically inhibiting sensory axons from entering the spinal cord resulted in the absence of sensory OLCs. Interestingly, this subset of oligodendrocytes displays stable sheaths, but does not turn on *myelin basic protein,* a hallmark of the myelination program, within the timeframe of these experiments which includes timepoints at 5 and 15 days post fertilization. In addition to being molecularly distinct, these sensory OLCs are not replaced by surrounding oligodendrocyte populations following laser ablation, suggesting a unique identity. Future work will be needed to determine the fate and maintenance of these specialized non-myelinating OLCs and better understand their function.

#### CNS-resident microglia emigrate into the PNS after spinal root injury

**Fig. 2.**
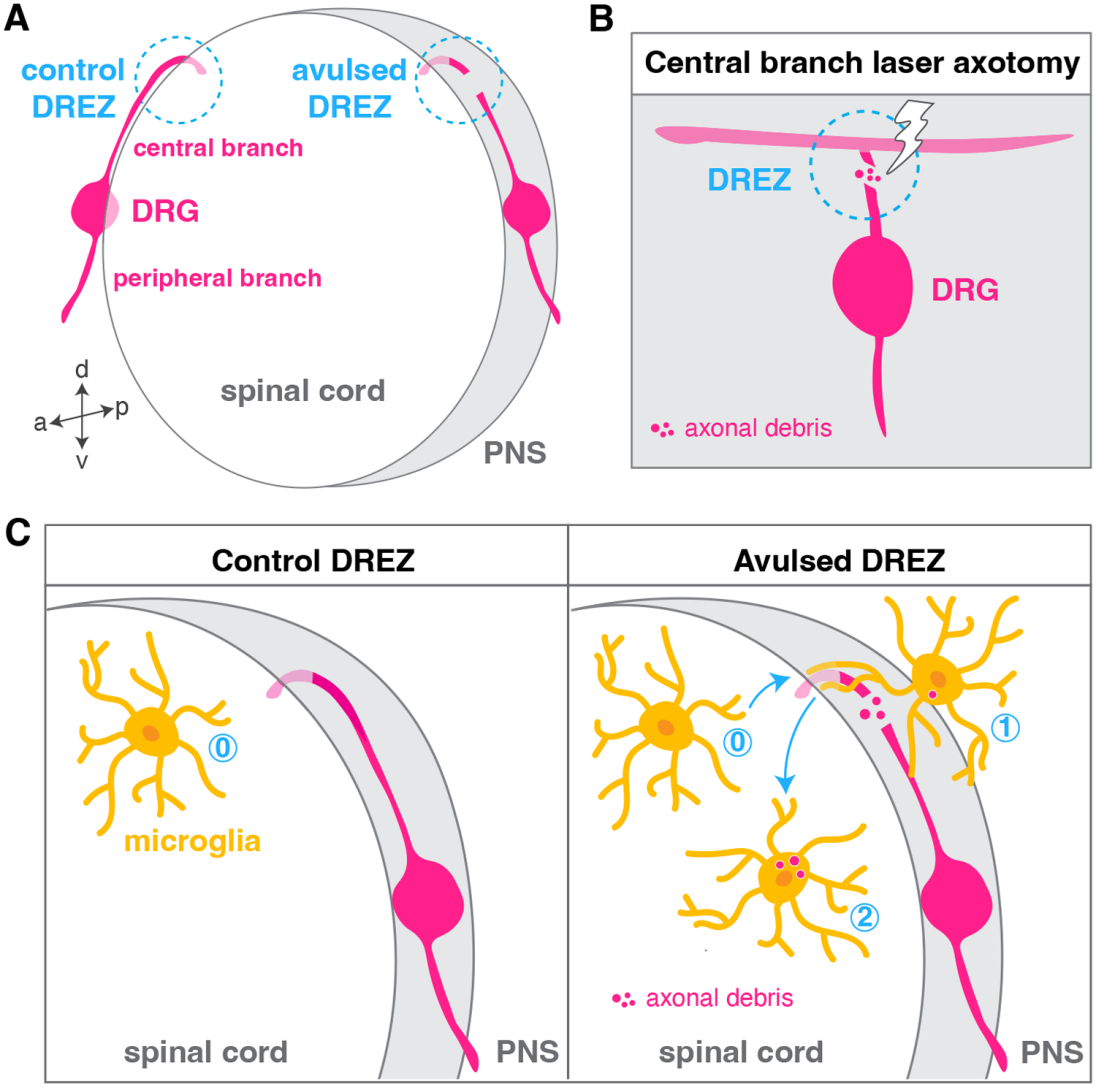
**Microglia exit the CNS in a model of spinal root avulsion.** (A) Diagram of a cross section of the spinal cord showing the intact peripheral and central branches of the dorsal rot ganglion (DRG) at a control DREZ, and an injured central branch at an avulsed DREZ. (B) Nitrogen-pulsed laser axotomy of the central branch of the DRG mimics spinal root avulsion. (C) Microglia are found exclusively inside the CNS (0) at the control DREZ. In the avulsed DREZ, microglia are observed exiting the spinal cord and phagocytose cell debris in the PNS (1). Almost half of these emigrated microglia return to the CNS over the next 24 h, carrying peripheral debris engulfed in their cytoplasm (2).

In another study, Green et al. showed that glial cells do more than communicating through the spinal cord entry zone. In a laser axotomy-mediated spinal root avulsion that models an obstetrical brachial plexus injury, a condition affecting newborns at a rate of 0.4 to 4 per 1000 births, the authors observed CNS-resident microglia migrate into the PNS at the DREZ and phagocytose peripheral debris (Fig. 2) (Green et al., 2019). In most examples of either CNS-located peripheral cells or PNS-located CNS cells, the ability of the ectopically emigrated cells to return to their respective compartment has not been documented. However, Green and collaborators demonstrated that once in the PNS, microglia could return to the CNS loaded with peripheral debris and were ultimately found in areas that include the spinal cord and the brain (Fig. 2). While these emigrated microglia often go back to the CNS, they return in an altered state reminiscent of activated disease-associated microglia (Green et al., 2019). Together, this work reveals that microglia, the professional phagocytes of the CNS, can function outside of the half of the nervous system in which they reside, and raises new questions about the short- and long-term consequences that these altered microglia might have for the proper function of neural circuits.

### The motor exit point

#### CNS-derived glia function in the PNS

In the 1980s, two independent studies performed in the rat and amphibian embryonic nervous systems suggested that non-neuronal cells emerging from the ventral root might guide motor axons to exit at the correct position and toward the periphery (Nordlander et al., 1981; Hockfield and McKay, 1985). Several decades later, with the emergence of high-resolution light microscopy and *in vivo* imaging of transgenic reporter lines, developmental biologists have identified some of these CNS-derived peripheral cells and determined their function.

Anatomical evidence in vertebrates and insects has been suggesting that peripheral glia play a role in guiding axons at the transition zones between the CNS and PNS, and that some of these cells have a central origin. To test the role of centrally derived peripheral glia in axon guidance, Sepp et al. conducted experiments using the *Drosophila* model system. After genetically eliminating peripheral glia during embryonic development, the authors observed abnormal patterns of motor axons exiting the ventral nerve cord, the insect analog of the vertebrate spinal cord. This study concluded that centrally derived peripheral exit glia prefigure the CNS/PNS transition zone and guide motor axons as they traverse this region (Sepp et al., 2001).

Consistent findings have been described in zebrafish and mouse, where spinal-cord-derived perineurial glia also guide motor axons into the PNS as they exit the spinal cord at MEP transition zones (Kucenas et al., 2008; Clark et al., 2014). These studies revealed the central origin of Nkx2.2^+^ perineurial glia, a subset of peripheral glia that contributes to the formation of the perineurium ensheathing peripheral spinal nerves by associating to one another via tight junctions. Genetic ablation of perineurial glia by using antisense morpholino oligonucleotides targeting *nkx2.2a* or a conditional null allele of the *Nkx2.2* gene in zebrafish and mouse respectively, demonstrated that perineurial glia play an important role in restricting motor neuron position within the spinal cord in addition to regulating motor axon fasciculation. Results from the zebrafish study showed that genetic ablation of *nkx2.2a^+^* cells lead to motor axons exiting the spinal cord ectopically and motor nerves were defasciculated (Kucenas et al., 2008). Because the *nkx2.2a* morpholino approach prevents perineurial glial specification as well as disrupting other lateral floor plate cells, future studies will be needed to address whether perineurial glia and/or other *nkx2.2^+^* cells such as their spinal cord-located precursors prefigure the motor exit point and guide motor axons into the PNS. The mammalian study later demonstrated that loss of Nkx2.2^+^ perineurial cells resulted in ectopic motor neuron cell bodies located outside of the spinal cord (Clark et al., 2014). Whether perineurial cells constrain motor neurons to the spinal cord from their peripheral location or from inside the spinal cord remains to be elucidated. Together, these findings illustrate the selective permeability of the spinal cord ventral transition zone and highlight the conserved role of centrally derived peripheral glia in shaping and maintaining the nervous system boundaries across divergent species.

#### Motor exit point glia restrict OLCs to the spinal cord

**Fig. 3.**
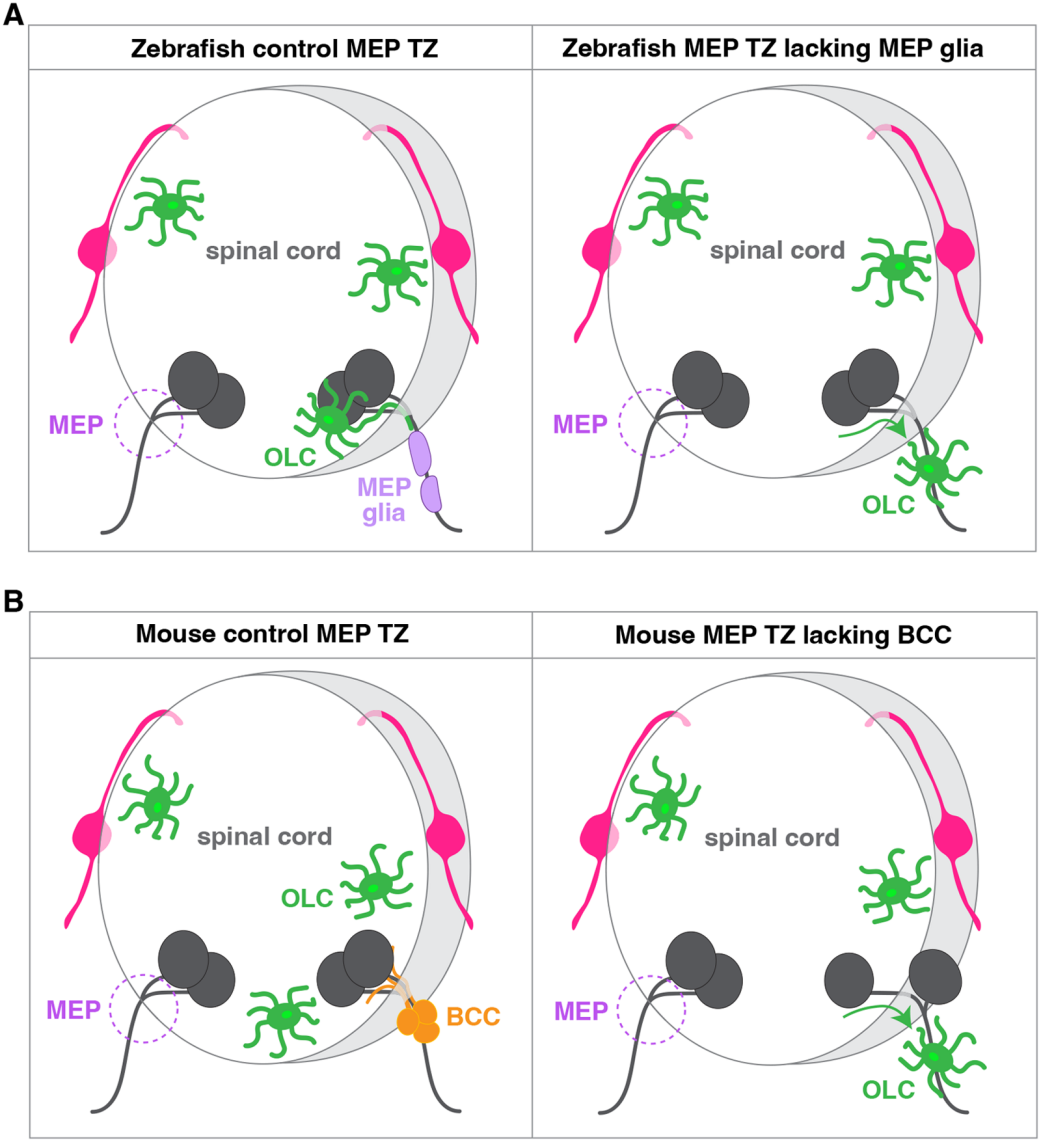
**Boundary cells restrict CNS components to the spinal cord at motor exit points.** (A) In zebrafish, motor exit point (MEP) glia (light purple) restrict oligodendrocyte lineage cells (OLC, green) to the spinal cord at control MEP transition zones (TZ). Upon genetic or laser ablation of MEP glia, OLCs migrate onto spinal motor nerves in the PNS. (B) In mouse, boundary cap cells (BC cells, orange) constrain CNS components to the spinal cord at MEP transition zones. In the absence of BC cells, central nervous system glia and motor neurons are found in the PNS.

While spinal-cord-located OLCs have the ability to survey the PNS by extending processes through MEP transition zones in physiology, oligodendrocyte cell bodies and myelin sheaths can be found in the PNS upon perturbation. Previously unknown glial cells named motor exit point (MEP) glia, were recently described at motor nerve roots (Smith et al., 2014). These glia originate in the lateral floor plate of the spinal cord, and migrate into the PNS at MEP transition zones, prior to OLC migration, using molecular mechanisms traditionally associated with neural crest-derived cells (Fontenas and Kucenas, 2021). Our work shows that MEP glia restrict OLCs to the spinal cord by contact-mediated repulsion and that pharmacological, genetic and laser ablation of MEP glia results in the presence of OLCs along spinal motor nerves in the PNS by 3 days post fertilization (Fig. 3A) (Smith et al., 2014; Fontenas et al., 2019; Fontenas and Kucenas, 2021). These reports also demonstrate that MEP glia myelinate motor axons, occupying axonal territory between oligodendrocytes and Schwann cells, and shed light on an unexplored source of myelinating glial cells conveniently located at the interface of the CNS and the PNS. Future studies further investigating the temporal regulation of glial cell migration at nervous system transition zones will define whether the CNS/PNS interface is permeable throughout life or only permeable during critical periods of embryonic development.

#### Boundary cap cells constrain CNS cells to the spinal cord

In chick and mouse embryonic development, a population of glial cells known as boundary cap (BC) cells, associates with ventral roots and contributes to the establishment of the CNS/PNS transition zone (Topilko et al., 1994; Niederlander and Lumsden, 1996). BC cells are transient neural-crest-derived cells that reside at the DREZ and at the MEP transition zone early in embryonic development, and eventually give rise to plethora of cell types, including Schwann cells (Maro et al., 2004; Gresset et al., 2015). In addition to being a source of peripheral cells, BC cells function to constrain CNS cells to the spinal cord (Fig. 3B) (Vermeren et al., 2003; Bron et al., 2007; Mauti et al., 2007; Coulpier et al., 2010). Surgical ablation of BC cells results in the transgression of the MEP transition zone by motor neuron cell bodies (Vermeren et al., 2003), and inactivation of the BC and Schwann cell marker Krox20 leads to the ectopic presence of OLCs and astrocytes in the PNS (Coulpier et al., 2010).

Fate mapping studies based on the expression of Krox20 (also known as early growth response protein 2, Egr2) and Putative Serine Protease 56 (Prss56) reported that BC cells migrate from the neural crest and arrive at MEP transition zones at E10.5 in mouse embryos, and were last observed at E16.5, when they contribute to the production of Schwann cells and other derivatives (Ghislain et al., 2002; Maro et al., 2004). By using Cre recombinase-based fate mapping and staining methods such as *in situ* hybridization in fixed tissue, discrete populations of BC cells might have escaped detection. A more recent piece of data illustrating BC cells extending long projections into the spinal cord at MEP transition zones brings a new perspective and does not rule out the possibility of a CNS origin for a subset of BC cells, reappraising the traditional lineage of this understudied glial cell type (Radomska and Topilko, 2017). The technical ability to identify all individual BC cells from their specification until they undergo their characteristic differentiation will be essential for their continuous visualization and lineage tracing in future studies.

#### Neural crest cells enter the spinal cord during early development

**Fig. 4.**
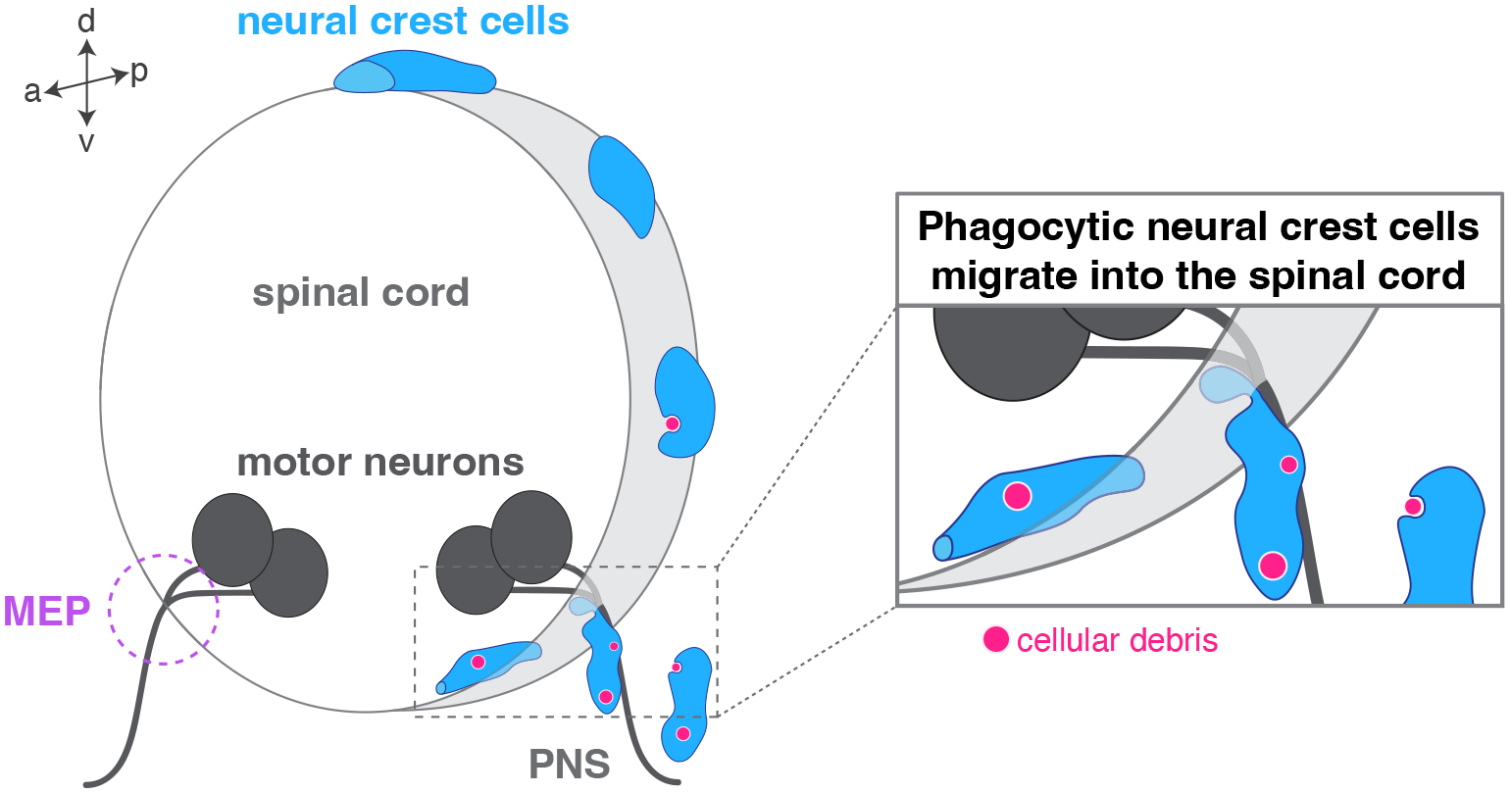
**Phagocytic neural crest cells enter the spinal cord through motor exit point transition zones during early development.** Neural crest cells (blue) migrate along the zebrafish neural tube and phagocytose PNS debris (magenta) until 36 h post fertilization. These non-professional phagocytes are occasionally found entering the spinal cord through motor exit point (MEP) transition zones and engulf debris in the CNS.

Such tools have recently contributed to the identification of a novel role for a traditional neural-crest-derived cell population. During nervous system development, programmed cell death naturally occurs in both the CNS and the PNS to regulate cell number and tissue sculpturing. In a study using live imaging in larval zebrafish, it was discovered that migratory neural crest cells respond quickly to dying cells and engulf cellular debris around the neural tube until 36 h post fertilization, which corresponds to the arrival of professional phagocytes (Zhu et al., 2019). In this study, Zhu and collaborators also showed that these phagocytic neural crest cells are capable of entering the spinal cord at MEP transition zones and clearing debris in the CNS during early development (Fig. 4). These findings are consistent with the study by Smith et al. that demonstrates that peripheral glial precursors transgress the PNS/CNS boundary at MEP transition zones to enter the neural tube prior to the maturation of radial glial endfeet that delineate the edge of the spinal cord (Smith et al., 2016). Together, these studies emphasize the synergy of multiple glial subtypes that allows for precisely regulated transgression of the MEP transition zone during embryonic development.

## Part III: toward applications in regenerative medicine

Recent work has demonstrated that the boundaries of the nervous system are a dynamic and plastic interface, and we are now starting to understand the cellular and molecular mechanisms underlying bidirectional cell migration across these barriers. Transition zones are unique regions where central and peripheral tissue meet and where CNS and PNS glial cells occupy non-overlapping territories. These territories are well defined along myelinated spinal axons, where oligodendrocytes produce layers of fatty membrane to myelinate the central portion of the fibers, and Schwann cells and MEP glia myelinate the peripheral segments. Damage to myelin sheaths (e.g. spinal cord injury, multiple sclerosis) results in axonal degeneration and loss of neuronal function and can be restored through a repair mechanism called remyelination. In the CNS, remyelination involves the presence of healthy OPCs and their differentiation into mature myelin-forming oligodendrocytes.

By taking advantage of powerful *in vivo* imaging in zebrafish, a recent remyelination study demonstrated that new oligodendrocytes exhibit more abundant and accurate myelin regeneration than those that survive demyelination (Neely et al., 2022). In a demyelination model where heat-sensitive oligodendrocyte myelin sheaths expressing mammalian transient receptor potential vanilloid 1 (TRPV1) were damaged by capsaicin, the authors showed that CNS remyelination by surviving oligodendrocytes is limited and mistargeted. Another recent demyelination study using a mouse model of multiple sclerosis revealed that surviving oligodendrocytes extend but rapidly lose new processes, and that they inefficiently form new myelin sheaths (Mezydlo et al., 2023). In the latter study, cortical demyelination was induced by stereotactic injection of the pro-inflammatory cytokines interferon-γ and tumor necrosis factor-α in the somatosensory cortex of mice that had been previously immunized with myelin-oligodendrocyte glycoprotein. Together, these elegant studies suggest that repairing CNS demyelinating lesions with new cells is a more efficient and more sustainable strategy.

Unlike the CNS, the PNS has the innate ability to regenerate and remyelinate mainly because of the contribution and plasticity of Schwann cells, which can dedifferentiate (Kumar et al., 2007; Johnston et al., 2016; Jessen and Arthur-Farraj, 2019). Therefore, taking advantage of the potential of Schwann cells to promote regeneration and remyelination in the CNS has become an innovative strategy for many research groups and Schwann cells are now thought of as promising rescuers of central demyelination (Zujovic and Van Evercooren, 2012; Garcia-Diaz and Baron-Van Evercooren, 2020; Chen et al., 2021).

Although spontaneous invasion of the CNS by Schwann cells was described in the demyelinated spinal cord and in rodent mutants where oligodendrocytes fail to make myelin such as the *taiep* and myelin deficient (*md*) rats, their presence is mostly limited to discrete areas near the spinal dorsal root entry zone and motor exit points (Blakemore et al., 1977; Yamamoto et al., 1991; Duncan and Hoffman, 1997; Garcia-Diaz et al., 2019). In these mutants, Schwann cell invasion was not detected until 6 to 8 weeks of age, and in the most severe, lethal phenotypes, was often preceded by the death of the animal (Duncan and Hoffman, 1997).

Numerous studies have focused on using Schwann cells to repair myelin lesions in the adult CNS. However, while Schwann cells efficiently remyelinate CNS axons and restore axonal function, they do not migrate far and subsequently only remyelinate limited areas within the spinal cord. This poor migration into the spinal cord has been investigated both *in vitro* and *in vivo* over the past decades (Bachelin et al., 2009; Cattin et al., 2015; Chaudhry et al., 2017; Garcia-Diaz et al., 2019). Results from a study using rodent Schwann cells suggest that in addition to the inhibitory interactions between Schwann cells and the glia limitans, CNS myelin components such as myelin-associated glycoprotein also repel Schwann cells and induce their death (Chaudhry et al., 2017). Contemporary work from Church and collaborators revealed that Schwann cell infiltration and remyelination of CNS lysolecithin-induced demyelinating lesions can be facilitated by macrophage activation (Church et al., 2017). The *in vivo* and *ex vivo* study led by Garcia-Diaz et al. not only revealed that Schwann cells use the vascular scaffold as their preferred route to infiltrate the demyelinated spinal cord and override the central inhibition created by CNS myelin, but also identified the signals regulating this migration (Garcia-Diaz et al., 2019). The authors showed that following the graft of Schwann cells into the lesioned spinal cord, EphrinB3 present in oligodendrocyte sheaths repelled Schwann cells away from CNS myelin via EphA4/B receptors, and in turn, increased Schwann cell adhesion to perivascular fibronectin and directed their migration along CNS blood vessels. This study elucidates the interplay between oligodendrocytes and Schwann cells that occurs in the early repair of spinal cord lesions and expands the toolbox for potential pharmacological applications to improve CNS remyelination.

## Conclusion

The past two decades have shed light on molecular mechanisms that regulate Schwann cell migration into the spinal cord as well as oligodendrocyte progenitor cell migration into the PNS, and have paved the way for the development of innovative therapeutical approaches for the treatment of neurodegenerative disorders and injuries (Smith et al., 2014; Morris et al., 2017; Fontenas et al., 2019; Garcia-Diaz et al., 2019; Fontenas and Kucenas, 2021). Future studies will be needed to further explore myelinating glial cell migration, myelination, and survival in the other half of the nervous system. The discovery of additional molecular mechanisms will allow the glial biology field to selectively target and boost myelin-forming cell migration at nervous system transition zones toward an improvement of remyelination and clinical recovery.
